# Synergistic Antifungal Effect of Fluconazole Combined with Licofelone against Resistant *Candida albicans*

**DOI:** 10.3389/fmicb.2017.02101

**Published:** 2017-11-07

**Authors:** Xinning Liu, Tao Li, Decai Wang, Yilei Yang, Wenwen Sun, Jianqiao Liu, Shujuan Sun

**Affiliations:** ^1^Department of Clinical Pharmacy, Taishan Medical University, Taian, China; ^2^Department of Microbial and Biochemical Pharmacy, School of Medicine and Pharmacy, Ocean University of China, Qingdao, China; ^3^Intensive Care Unit, Qianfoshan Hospital Affiliated to Shandong University, Jinan, China; ^4^Department of Pharmacy, Qianfoshan Hospital Affiliated to Shandong University, Jinan, China; ^5^General Practice, Shandong Provincial Hospital, Jinan, China

**Keywords:** biofilms, cyclooxygenase (COX), drug resistance mechanisms, enzyme inhibitor, phospholipase

## Abstract

*Candida albicans* (*C. albicans*) is one of the important opportunistic fungal pathogens that is closely associated with disseminated or chronic infections. The objective of this study is to evaluate the synergistic antifungal effect of licofelone, which is dual microsomal prostaglandin E2 synthase/lipoxygenase (mPGES-1/LOX) inhibitor in combination with fluconazole against *C. albicans*. Here our results showed that licofelone (16 μg/mL) can synergistically work with fluconazole (1 μg/mL) against planktonic cells of fluconazole-resistant *C. albicans.* The two-drug combination inhibited the *C. albicans* biofilm formation over 12 h, and reduced the expression of extracellular phospholipase genes, biofilm-specific genes and RAS/cAMP/PKA pathway related genes. In addition, the two-drug combination inhibited the transition from yeast to hyphal growth form, and decreased the secreted aspartyl proteinase activity, while not affecting the drug efflux pumps activity. *Galleria mellonella* model was also used to confirm the antifungal activity of the drug combination *in vivo*. This study first indicates that the combination of fluconazole and licofelone has synergistic effect against resistant *C. albicans* and could be a promising therapeutic strategy for the antifungal treatment.

## Introduction

*Candida albicans* is one of the important fungal pathogens that forms biofilm on the surface of catheters and other medical devices. It is also believed to be mainly implicated in disseminated or chronic fungal infections in ill and immunocompromised individuals (Carrillo-Munoz et al., 2006). Currently, the antifungal drugs used in a clinical practice include azoles, amphotericin B, and echinocandins. Amphotericin B is a potent antifungal agent against an array of yeast and filamentous fungal pathogens, however, the application is limited by the significant toxicities such as renal toxicity, infusion reactions, and hepatotoxicity (Clements and Peacock, 1990). Both amphotericin B and echinocandin drugs also have minimal gastrointestinal absorption and are only available as parenteral formulations, while fluconazole, one of the commonly used first-line drug of the azole family in clinical prevention and treatment of Candida infections, is readily absorbed with high bioavailability (Nett and Andes, 2016). The wide-spread use of antifungal drugs has increased the incidence of candida-resistance, ultimately leading to refractory fungal infections (Chen et al., 2012; Pfaller et al., 2015). In the United States, fluconazole-resistance has caused significant additional hospitalization costs and deaths (Sagatova et al., 2015). Therefore, seeking novel agent combined with fluconazole against resistant *C. albicans* is a urgent need.

Licofelone, a dual mPGES-1/LOX inhibitor is the terminal enzyme in the biosynthesis of Prostaglandin E2 (PGE_2_). Previous studies have noted that PGE_2_, as the final product of arachidonic acid metabolic pathway can mediate several biological phenomena (Harizi et al., 2008). It is also associated with the ability of *C. albicans* to switch between yeast and hyphal growth forms. Recent studies indicated that PGE_2_ synthesized from host and fungal can promote cell adhesion, germ tube formation and enhance fungal resistance in *C. albicans* (Kalo-Klein and Witkin, 1990; Noverr et al., 2001). RAS/cAMP/PKA pathway is important for mediating the transition from yeast to hyphal growth form in *C. albicans* and is known to regulate the expression of many biofilm-specific genes including *EFG1*, *BCR1*, *ALS1*, *ALS3*, and *HWP1* (Hogan and Sundstrom, 2009). Latest study shows that PGE_2_ increased cAMP level, activated PKA in *C. albicans*-infected macrophages, and therefore stimulated *C. albicans* germ tube formation (Yun et al., 2016). Based on this, we hypothesize that the regulation of PGE_2_ influencing biofilm formation may be associated with the RAS/cAMP/PKA pathway. In several studies, the synergistic effects of cyclooxygenase (COX) inhibitors combined with antifungal drugs against *C. albicans* biofilm and planktonic cells have been observed. These potential antifungal activities are supposed to be associated with the regulation of PGE_2_ production (Alem and Douglas, 2004; de Quadros et al., 2011; Rusu et al., 2014; Liu et al., 2016). A dual mPGES-1/LOX inhibitor, licofelone can suppress PGE_2_ formation (Koeberle et al., 2008) and has recently succeeded in reaching the required criteria in phase III clinical trials for osteoarthritis (Payandemehr et al., 2015), but no researches in regards with its antifungal activity and mechanism against planktonic and biofilm cells of *C. albicans* have been carried out so far.

In this study, we first evaluated the *in vitro* efficacy of licofelone alone and in combination with fluconazole against *C. albicans* by the checkerboard microdilution method, and observed their antimicrobial effects on biofilm formation. In addition, the toxicity of *C. albicans* treatment was investigated by *Galleria mellonella* model *in vivo*, survival analysis, and histology. The fungal burden determination was used to confirm the antifungal effect of combined treatment during *C. albicans* infections. Related antifungal mechanisms were also explored in this study. Various virulence factors, other than biofilm formation, have been contributed to *C. albicans* pathogenicity in the hosts, such as secreted aspartyl proteinase and phospholipase activity (Garcia-Vidal et al., 2013; Cui et al., 2015). Aspartyl proteinase secreted from *C. albicans* is directly related to virulence properties such as adhesion, tissue invasion and immune evasion (Braga-Silva and Santos, 2011). *C. albicans* produces phospholipases, which can destroy cell membranes during host cell invasion (Ghannoum, 2000). It has been shown that phosphatidylcholine-specific phospholipase D1 is important for yeast to hyphal transitions under certain conditions in *C. albicans* (Hube et al., 2001). The gene expression levels including RAS/cAMP/PKA pathway related genes (*RAS1*, *CYR1*, *TPK2*), biofilm-specific related genes (*EFG1*, *BCR1*, *ALS1*, *ALS3*, *HWP1*), and secreted aspartyl proteinase (*SAP*) genes (*SAP1*, *SAP2*, *SAP3*, *SAP4*) were assessed by RT-PCR. The morphogenesis of *C. albicans* was also observed by fluorescence microscope. We also detected the effect of the two-drug combination on extracellular phospholipase activities by egg yolk agar method and measured the drug efflux pumps activity by rhodamine 6G assay.

## Materials and Methods

### Strains and Media

Eight clinical isolates were obtained from Shandong Qianfoshan Hospital in China. Six resistant strains, *Candida albicans* (CA10, CA16), *Candida glabrata* (CG2, CG3) and *Candida parapsilosis* (CP2, CP3); and two sensitive strains, *Candida albicans* (CA4, CA8) were used in this study. Their susceptibilities were determined according to Clinical and Laboratory Standards Institute document M27-A3 (Wayn, 2008) with *C. albicans* (ATCC 10231) as the quality control strain. The strains were maintained at -80°C and subcultured at least twice on the yeast–peptone–dextrose (YPD) solid medium at 35°C. Licofelone and fluconazole were purchased from Shanghai Boylechem Co., Ltd., China and Dalian Meilun Biotech Co., Ltd., China, respectively. The stock solution was prepared according to the manufacturer’s instructions. Briefly, fluconazole was dissolved in sterile deionized water at room temperature, and licofelone was dissolved in dimethyl sulfoxide (DMSO) with 0.2% Tween 80. Each stock solution was prepared at a final concentration of 2560 μg/mL and stored at -20°C until needed. RPMI1640 was used as a diluent medium for drugs and strains. The minimal inhibitory concentration (MIC) was defined as the lowest concentration of the drug that inhibited fungal growth by 80% (MIC80) compared with that of the growth control and sessile MIC (sMIC) was read as the lowest concentration that produced a 50% reduction in growth compared with that of the drug-free control. Before conducting the large number of experiments, the MIC and sMIC of which fluconazole and licofelone used alone in the experiments was first evaluated as a preliminary experiment.

### MIC Determination by Broth Microdilution Assays

The MICs of licofelone and fluconazole against *C. albicans* strains were determined by the broth microdilution method according to the Clinical and Laboratory Standards Institute (CLSI) standard M27-A3 (Lockhart et al., 2011). For the checkerboard assays (Kuykendall and Lockhart, 2016), the final concentration of fluconazole and licofelone used were 0.125–64 μg/mL and 2–128 μg/mL, respectively, for resistant candida strains (CA10, CA16, CG2, CG3, CP2, CP3); and 0.03125–16 μg/mL and 2–128 μg/mL, respectively, for sensitive candida strains (CA4, CA8). All drugs were diluted in 50 ml sterile RPMI 1640 medium. Fluconazole and licofelone were added to the wells in the second to eleventh columns and A through G lines of the 96-well plate, respectively. 100 μl of *C. albicans* cell suspensions (0.5∼2.5 × 10^3^ cells/mL) were then inoculated into each well of 96-well plates. Each wells were filled with RPMI-1640 to a final volume of 200 μl except the control plate. Negative controls were performed in 200 μl RPMI1640 in A12-H12 wells, and growth controls were performed in 100 μl RPMI1640 and 100 μl microorganisms in the H1 well. The plate was incubated at 35°C for 24 h. Both visual reading and optical density (OD) were performed to determine the MIC value (Bertout et al., 2011). OD was measured on the absorbance at 492 nm on a microplate reader. MIC endpoints were defined as the lowest concentration of drugs causing 80% decrease in viability compared to the drug-free control (MIC80). All experiments were performed in triplicate. Drug interactions were interpreted by the fractional inhibitory concentration index (FICI) model and the percentage of growth difference (ΔE) model. The FICI ≤ 0.5 represents synergy, FICI 0.5 < FICI ≤ 4 represents no interaction, and FICI > 4.0 represents antagonism (Odds, 2003). The ΔE model was defined by the following equation: ΔE = E_predicted_ - E_measured_. The E value was calculated by the data obtained directly from experiments. Statistically significant interactions of <100% were considered weak, those from 100 to200% were considered moderate, and those of >200% were considered strong, as previously described (Katragkou et al., 2015).

### sMIC Determination by Broth Microdilution Assays

*Candida albicans* biofilm was developed using a slightly modified method described above (Gu et al., 2016; Sun et al., 2017). The sMICs of fluconazole and licofelone against *C. albicans* (CA10, CA16, CA4, CA8) biofilms were tested. The biofilms were formed over three time intervals (8, 12, and 24 h) at 35°C by pipetting 100 ml of the standardized cell suspension (10^3^ cells/mL) into selected wells of 96-well plate. After each adhesion phase (8, 12, and 24 h), the cell suspensions were gently washed three times with sterile phosphate-buffered saline (PBS) and the planktonic yeast were removed. Fluconazole and licofelone were then added to the corresponding wells of 96-well plate in serially double-diluted concentrations. The final concentration of fluconazole and licofelone in wells was ranged from 1–512 μg/mL and 2-128 μg/mL for each isolate, respectively. The control wells were filled with 200 μl RPMI 1640 only. The plates were then incubated for another 24 h at 35°C in an orbital shaker. A colorimetric reduction assay was carried out with 2,3-bis-(2-methoxy-4-nitro-5-sulfophenyl)-2H-tetrazolium-5-carboxanilide (XTT) according to the protocol of Melo et al. The absorbance was measured with a microtiter plate reader at the absorbance of 492 nm, and the drug concentration that brought about a reduction in absorption by 50% contrasted to that in the control well was reported as the sMIC endpoint. Each test was performed in triplicate.

### Efficacy of Fluconazole and Licofelone in *G. mellonella* Infected with *C. albicans* by Survival Assay

*Galleria mellonella* in the final instars larval stage of development was used as previously described with some modifications (Mylonakis et al., 2005; Amorim-Vaz et al., 2015). Larvae were divided into four groups: growth control group (PBS only), fluconazole (160 μg/mL) treated group, licofelone (80 μg/mL) treated group, and fluconazole (160 μg/mL) with licofelone (80 μg/mL) treated group. Control group of larvae inoculated was studied in parallel in every infection investigation. The larvae were stored in wood shavings in petri dishes at 35°C in the dark condition for 2 days before the experiments. Larvae with dark spots or apparent melanization were excluded. Resistant *C. albicans* isolates (CA10) were grown overnight on liquid Sabouraud medium, and the inocula were suspended in PBS buffer supplemented with 20 μg/mL of ampicillin to prevent bacterial contamination. 10 μl of each larva suspension (5 × 10^6^ cells/mL) were injected of through the last left proleg using a 10-l syringe (Gaoge, China). The infected larvae were incubated at 35°C for 2 h, and then treated with different group of antifungal drugs. The death of larvae was monitored by visual inspection of their color (brown-dark brown) each 24 h for 4 days. The survival experiments were terminated at 4 days after infection. Each experiment groups contained 20 larvae (0.2–0.25 g in weight) and the experiment was repeated three times using larvae from different batches.

### Fungal Burden Determination

Fungal burden was determined by colony-forming unit (CFU) counts for 4 days after infection (Krezdorn et al., 2014). Larvae were divided into four groups: growth control group (PBS only), fluconazole (160 μg/mL) treated group, licofelone (80 μg/mL) treated group, and fluconazole (160 μg/mL) with licofelone (80 μg/mL) treated group. Each larva was infected with 5 × 10^6^ cells/larva of resistant *C. albicans* (CA10) and incubated at 35°C for 2 h, then treated with different drug. At every 24 h, 3 larvae from each group were selected with no discrimination, suspended in 1 ml of PBS-ampicillin, and gently homogenized for a few seconds. The mix was 10-fold diluted with PBS buffer and 10 μl of these dilutions were inoculated on the sabouraud agar plates. The plates were incubated at 35°C for 24 h and the number of CFUs was counted. The results were expressed as mean standard deviation (SD). Each experiment group contained 20 larvae (0.2–0.25 g in weight) and experiment was repeated three times using larvae from different batches.

### Histological Study

To evaluate the presence of resistant *C. albicans* (CA10) in tissue of *G. mellonella*, three larvae from different groups (infected only; and infected and treated with licofelone, fluconazole and licofelone-fluconazole) were collected at day 3 after infection. The larvae were preserved in 70% ethanol and cut into 8-μm-thick sections using a cryostat. The samples were dried naturally at room temperature for 2 days, stained with Periodic Acid Schiff reagent (PAS), and dehydrated with increasing concentrations of ethanol and xylol (70, 80, 90, 96, and 100%). Finally, the samples were fixed in neutral balata and dried naturally at room temperature for 2 days. The stained tissues were observed under an Olympus FSX100 fluorescence microscope with ×4.2 objectives. The experiment was repeated three times using larvae from different batches.

### Phospholipase Activity of *C. albicans* Treated with Licofelone

Phospholipase activity of resistant *C. albicans* (CA10) treated with different drugs was detected by egg yolk agar plate method with some modifications (Niewerth and Korting, 2001). The egg yolk medium consisted of 15 g/L peptone, 3 g/L beef extract ointment, 5 g/L NaCl, 15 g/liter agar, 10 g/L glucose, and 10% sterile egg yolk emulsion. Each culture with 10^7^ CFU/mL were treated with different drug group including fluconazole (1 μg/mL) group, licofelone (16 μg/mL or 32 μg/mL) group, fluconazole (1 μg/mL) combined with licofelone (16 μg/mL or 32 μg/mL) group, and growth control (drug-free) group, transferred into separate eppendorf tubes, and then incubated at 35°C for 24 h. Then, 10 μl suspension of each tubes were inoculated onto the medium, the plates were incubated at 35°C for 72 h. The diameter of the precipitation zone (a) and the diameter of the precipitation zone plus the diameter of the colony (b) were measured. Phospholipase zone (Pz) was designated as Pz = a/b, as described above. According to this definition, phospholipase index was scored and categorized as follows: negative (Pz = 1); very low (Pz = 0.90 to 0.99); low (Pz = 0.80 to 0.89); high (Pz = 0.70 to 0.79), and very high (Pz ≤ 0.69), as previously reported (Price et al., 1982). Each experiment was tested in triplicate and the phospholipase activity value was recorded as the average of the 3 measurements.

### Real-Time Quantitative PCR

mRNA expression of secreted aspartyl proteinase-related genes (*SAP1*, *SAP2*, *SAP3*, and *SAP4*), RAS/cAMP/PKA pathway related genes (*RAS1*, *CYR1*, *TPK2*), and biofilm formation related genes (*EFG1*, *BCR1*, *HWP1*, *ALS1*, *ALS3*) was measured by RT-PCR. Resistant *C. albicans* (CA10) cells were grown to mid-log phase in RPMI-1640 medium at 35°C after treatment with fluconazole (1 μg/mL) alone group, licofelone (16 μg/mL) alone group and fluconazole (1 μg/mL) combined with licofelone (16 μg/mL) group. Cultures without drugs were served as controls. Total RNA was extracted and isolated from cells using RNA pure yeast kit (DNase I) (CWBiotech, Beijing, China). A diluted RNA was reverse transcribed to cDNA using first-strand cDNA synthesis Super Mix kit (CWBiotech) at 42°C for 30 min following 85°C for 5 min according to the manufacturer’s instructions. RT-PCR reactions were performed using cDNA, ultra SYBR mixture (with ROX) (CWBiotech), and primers with an ABI ViiA 7 (Applied Biosystems) sequence detection system (CWBiotech). An aliquot of 25 μl PCR mix was used for each gene and the cycling conditions were 95°C for 10 min, followed by 40 cycles of 95°C for 15 s and 60°C for 1 min. ACT1 was found to be performed well under all experimental conditions, therefore, in this study, ACT1 was chosen to be suitable for use as an endogenous control gene (Cao et al., 2012; Bu et al., 2016). Primers used in this study are in Supplementary Table S1. The experiment was repeated on 3 independent occasions.

### *C. albicans* Morphogenesis Analysis

The morphogenesis analysis was carried out as described before (Pierce et al., 2015). The cells of resistant *C. albicans* (CA10) were grown overnight at 30°C in RPMI1640 and treated with different drugs including growth control (drug-free) group, fluconazole (1 μg/mL) group, licofelone (16 μg/mL) group, and fluconazole (1 μg/mL) combined with licofelone (16 μg/mL) group in the well plates. The treated suspensions were then examined by fluorescence microscope to determine the percentage of hyphal formation. The experiment was repeated on 3 independent occasions.

### Rhodamine 6G Efflux Assays

The determination of the functional activity of drug efflux pumps affected by licofelone was carried out (Sun et al., 2015). Briefly, resistant *C. albicans* (CA10) cells were shaken overnight at 35°C in YPD broth and then washed three times with PBS. The cells were harvested by centrifugation and adjusted to 1 × 10^7^ cells/ml in PBS. In order to assess the efflux of Rh6G, we set 2 groups in this experiment: growth control group (drug-free) and licofelone (16 μg/mL) group. Rhodamine 6G (final concentration of 10 μM) was added to each cell suspension. After incubating 55 min at 35°C, *C. albicans* cells were collected by centrifuging at 3,000 rpm for 5 min, washed three times with PBS, and resuspended with PBS containing 5% glucose. Five hundred microlliter of each sample were withdrawn at every 10 min. The mean fluorescent intensity (MFI) was immediately determined using the flow cytometer with excitation at 488 nm and emission at 530 nm at each specific time intervals. The experiment was repeated on 3 independent occasions.

### Statistical Analysis

Data are presented as mean ± SEM. All experiments were performed in triplicate. Graphs production, data distribution and statistical analyses were performed using Graph Pad Prism 5. After ensuring data conformed to a normal distribution, before and after data transformation, analysis of variance (ANOVA) and *t*-tests were used to investigate significant differences between independent groups. The *G. mellonella* survival curves were analyzed by the Kaplan–Meier method, and fungal burdens were analyzed using a *t*-test to measure statistical differences between two independent groups. *P* < 0.05 was considered as statistically significant.

## Results

### MICs Determined by Broth Microdilution Assays

**FIGURE 1:**
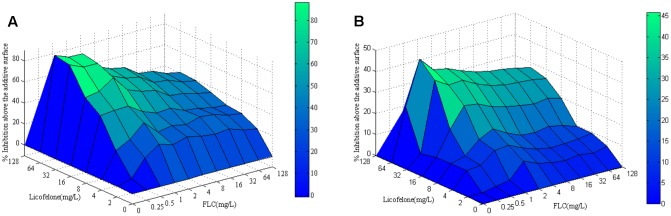
Drug interactions of fluconazole and licofelone are interpreted by the ΔE model. Three-dimensional plots of fluconazole combined with licofelone against *Candida albicans* were created by using MATLAB program. The concentrations of fluconazole and licofelone are depicted on the *x* axis and *y* axis, respectively, and the *E*-values obtained for each combination is depicted on the z axis to construct a three-dimensional (3D) graphic. Peaks above the 0 plane represent synergistic combinations. The color-coding bar on the right indicates that the closer to the top of the bar, the more effective the drug combination. **(A)** The ΔE model of *Candida albicans*10, **(B)** the ΔE model of *Candida albicans* 16.

The susceptibilities of 8 *Candida* isolates (CA4, CA8, CA10, CA16, CG2, CG3, CP2, CP3) were assessed under planktonic states. For all of the strains, the MIC of licofelone used alone was 128 μg/mL, and the MICs of fluconazole alone were ranged from 1 to 512 μg/ml (**Figures 1A,B**). Susceptibility assay showed that the combination of licofelone and fluconazole has strong synergistic antifungal effects against resistant *C. albicans*: the MIC_80_ of fluconazole alone against resistant *C. albicans* (CA10, CA16) were >512 μg/mL, whereas when combined with licofelone (16 μg/mL), the MICs of fluconazole were decreased to 1 and 0.5, respectively. These effects were also illustrated by the FICI *in vitro*: the FICI for the resistant *C. albicans* (CA10, CA16) strains is 0.127 and 0.250, respectively (**Table 1**), however, the FICI of sensitive *C. albicans* (CA4, CA8) and non-albicans (CG2, CG3, CP2, CP3) strains were all >0.5, indicating that the two-drug combination exhibited no antifungal activity against the sensitive *C. albicans* and non-albicans. These results were also interpreted by ΔE method.

**Table 1:** The combined antifungal effects of fluconazole combined with licofelone against *Candida albicans*.

Drug	Strains	MICs (μg/mL)				LA theory		BI theory
		MIC_A_	C_A_	MIC_B_	C_B_	FICI	IN	ΣSYN
Fluconazole + Licofelone	CA10 (R)	>512	1	128	16	0.127	SYN	2704%
	CA16 (R)	>512	0.5	128	32	0.250	SYN	1112%
	CA4(S)	1	0.5	128	64	1.000	NI	<100%
	CA8(S)	2	1	128	64	1.000	NI	<100%
	CG2 (R)	128	32	128	64	0.750	NI	<100%
	CG3 (R)	64	16	128	64	0.750	NI	<100%
	CP2 (R)	128	2	128	64	0.516	NI	<100%
	CP3 (R)	128	4	128	64	0.531	NI	<100%

*Eight isolates with different susceptibilities involved three Candida spp. CA, *Candida albicans*; CG, *Candida glabrata*; CP, *Candida parapsilosis*. MICs denote the MIC80 of each drug alone or in combination against the isolates and are shown as the median of three independent experiments; S, susceptible; R, resistant. The FICI value is the median of three independent experiments; FICI ≤ 0.5 for synergism, FICI > 4.0 for antagonism and 0.5 < FICI ≤ 4.0 for no interaction*.

### sMIC Determined by Broth Microdilution Assays

The susceptibilities of 4 *C. albicans* (CA4, CA8, CA10, CA16) isolates were assessed under biofilm states. For the resistant *C. albicans* (CA10, CA16) and sensitive *C. albicans* (CA4, CA8), the sMIC of fluconazole and licofelone used alone was 512 μg/mL and 128 μg/mL, respectively. The susceptibility test showed that licofelone (16 μg/mL) combined with fluconazole (1 μg/mL) had synergistic antifungal effects against *C. albicans* biofilms (FICI < 0.5) at both 8 and 12 h time point. However, after 12 h formation, the combined effect of fluconazole and licofelone on biofilm has gradually reduced. The combined antifungal effect was barely observed on the biofilm formed over 24 h with FICI > 1, suggesting that the two-drug combination has synergistic antifungal effect at the early stage, but not the mature biofilm. The following experiments were performed in resistant *C. albicans* (CA10) isolate due to its strong susceptibility to the combination of licofelone and fluconazole. The SMICs of combined antifungal drugs are shown in **Table 2**.

**Table 2:** Antifungal effect of fluconazole combined with licofelone against biofilm of resistant *Candida albicans*.

Strains	Times (h)	SMIC(μg/mL)				LA theory	
		MIC_A_	C_A_	MIC_B_	C_B_	FICI	IN
CA10 (R)	8	>512	2	>128	32	0.252	SYN
	12	>512	4	>128	32	0.254	SYN
	24	>512	256	>128	128	>1	NI
CA16 (R)	8	>512	4	128	16	0.133	SYN
	12	>512	4	128	32	0.258	SYN
	24	>512	256	128	128	>1	NI
CA4 (S)	8	>512	8	128	16	0.141	SYN
	12	>512	16	128	16	0.156	SYN
	24	>512	256	128	128	>1	NI
CA8 (S)	8	>512	4	128	8	0.070	SYN
	12	>512	8	128	16	0.141	SYN
	24	>512	256	128	128	>1	NI

*Time indicates the incubation period of biofilm formation. SMICs were read as the lowest concentrations that produced a 50% reduction in growth compared with that of the drug-free control. MIC_*A*_, The MICs of fluconazole used alone; MIC_*B*_, The MICs of licofelone used alone; C_*A*_, The MICs of fluconazole combined with licofelone; C_*B*_, The MICs of licofelone combined with fluconazole; IN, interaction; SYN, synergism; NI, no interaction. The FICI value is the median from three independent experiments. FICI ≤ 0.5 for synergism, FICI > 4.0 for antagonism and 0.5 < FICI ≤ 4.0 for no interaction*.

### Efficacy of in *G. mellonella* Infected with *C. albicans*

**FIGURE 2:**
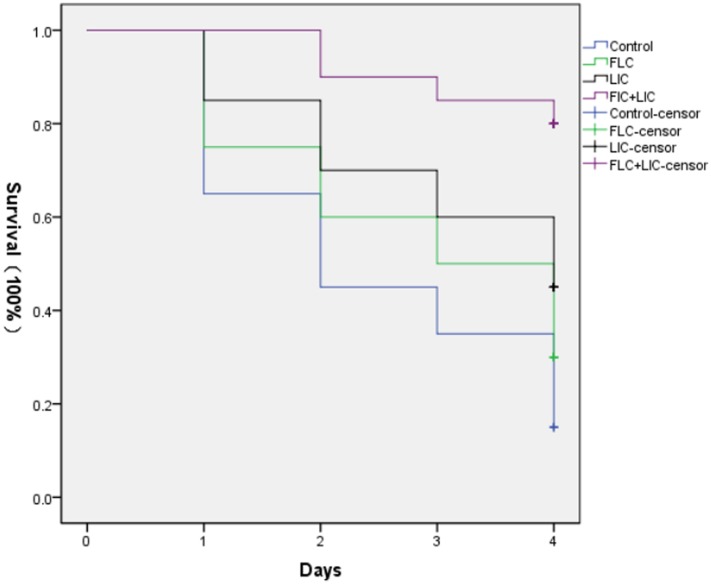
Survival curve of different treatment on *G. mellonella* infected with resistant *C. albicans* (CA10). The concentration of yeast cells was 5 × 10^6^ cells/larva. The curves were consisted growth control (PBS) group, fluconazole (160 μg/mL) group, licofelone (80 μg/mL) group and licofelone (80 μg/mL) combined with fluconazole (160 μg/mL) group. These four curves were put in the same coordinate system to compare the survival rates. ^∗^*P* < 0.05 compared to the control, fluconazole alone and licofelone alone group. The experiment was repeated on 3 independent occasions (*n* = 3). Values represent the means standard deviations from three replicates.

*In vivo* effect of fluconazole (160 μg/mL), licofelone (80 μg/mL), and their combination on infected *G. mellonella* treated was determined, and the melanization of *G. mellonella* infected with resistant *C. albicans* (CA10) was observed. The selection of drug concentration is not shown in this research. The results showed that after 2 days infection, all of the drug-treated groups showed attenuated melanization (**Figure 2**). Specifically, the two-drug combination group significantly reduced the melanization compared to the fluconazole group and was the highest survival rate (*P* < 0.05). After 4 day infection, the survival rate of larvae treated with fluconazole and combination group was higher than the growth control group by 1.3 and 4-fold, respectively. The drug combination group was also highest (*P* < 0.05). The survival rate of larvae treated with licofelone alone group was a little higher than that of larvae treated with fluconazole group alone, that may be explained by the fact that the antifungal activity of licofelone alone is stronger than fluconazole.

### Fungal Burden Determination

**FIGURE 3:**
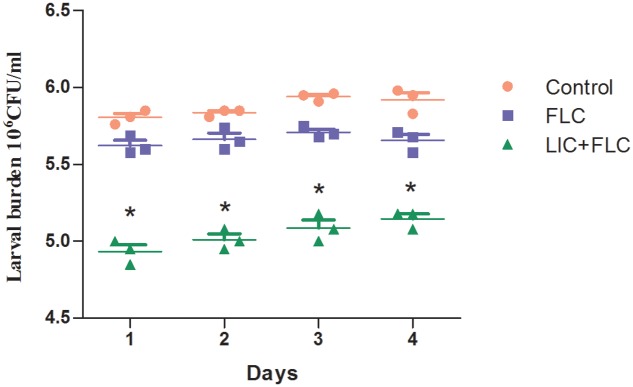
Effect of drug combination on larval burdens of resistant *C. albicans* (CA10). All larvae were infected with 5 × 10^6^ cells/larva *C. albicans*, the treatment consisted of control (PBS alone) group, fluconazole (160 μg/mL) alone group, and fluconazole (160 μg/mL) combined with licofelone (80 μg/mL) group. ^∗^*P* < 0.05 compared to the control, and fluconazole alone group. The experiment was repeated on 3 independent occasions (*n* = 3). Values represent the means standard deviations from three replicates.

After 3 days of infection, the fungal burden was determined by recovering yeast cells from the larvae infected with resistant *C. albicans* (CA10). The number of CFUs in larvae were increased over the time of infection (**Figure 3**). Infected larvae that were treated with fluconazole (160 μg/mL) in combination with licofelone (80 μg/mL) exhibited lower fungal burden than fluconazole group. The two-drug combination significantly decreased CFU number by almost 4-fold compared to the control group (*P* < 0.05). The CFU number of the control group was also slightly decreased, which may be caused by hemocytes in larvae.

### Histopathology Study

**FIGURE 4:**
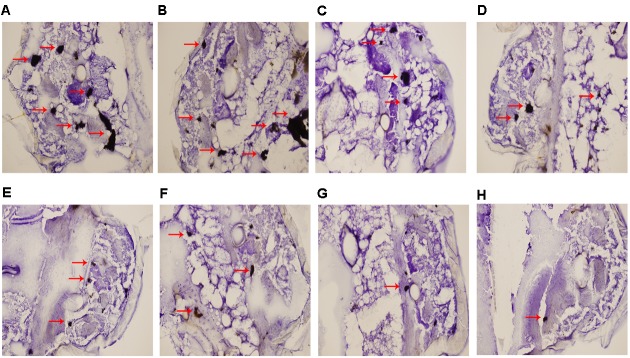
Histopathology study of infected *G. mellonella* treated with different drugs. After 72 h of infection, larvae infected with 5 × 10^6^ cells/larva of resistant *C. albicans* (CA10) were processed for histopathology as described. **(A,B)** Untreated controls group; **(C,D)** larvae treated with fluconazole (160 μg/mL) group; **(E,F)** larvae treated with licofelone (80 μg/mL) group; **(G,H)** larvae treated with the combination of fluconazole (160 μg/mL) and licofelone (80 μg/mL) group. The experiment was repeated on 3 independent occasions.

Histopathologic staining of larvae infected with resistant *C. albicans* (CA10) and treated with different drugs was performed at 3 day post infection. The differences in shape and cytoplasmic staining were detected. Both yeast and filament forms of *C. albicans* formed clusters in infected larvae. In the treatment with antifungal drugs, the formation of a majority of yeasts was decreased in the combination of fluconazole (160 μg/mL) and licofelone (80 μg/mL) group (**Figures 4G,H**). In addition, licofelone alone group (**Figures 4E,F**) decreased more yeast cells than fluconazole alone group (**Figures 4C,D**). The growth control (treatment with the PBS alone) group (**Figures 4A,B**) has higher levels of infection than any other group. The mean size of the infected areas was smaller in the combination group compared to the fluconazole group.

### Effect of Licofelone on Extracellular Phospholipases Activity of Resistant *C. albicans*

The extracellular phospholipase activity of resistant *C. albicans* (CA10) was measured (**Table 3**). There were no obvious differences in average Pz between control group, fluconazole (1 μg/mL) group, and licofelone (16 μg/mL) group. The precipitation zone of the combination of fluconazole (1 μg/mL) and licofelone (16 μg/mL) group was significantly increased to 0.91 compared to the control group (*P* < 0.05). No precipitation zone was observed in licofelone (32 μg/mL) combined with fluconazole group (*P* < 0.05) and licofelone used alone (32 μg/mL) group.

**Table 3:** Extracellular phospholipase activity of *C. albicans* treated with licofelone and fluconazole.

Group (μg/mL)	Precipitation zone	Phospholipase activity
Control	0.61	High
Fluconazole (1)	0.62	High
Licofelone (16)	0.76	High
Fluconazole + Licofelone (16)	0.91^a^	Very low
Licofelone (32)	NZ^b^	
Fluconazole + Licofelone (32)	NZ^b^	

*The precipitation zone represents the ratio of the diameter of the colony to the cloudy zone plus colony diameter. ^a^*P* < 0.05 compared to the control. ^b^NZ, no zone of precipitation*.

### Effect of Fluconazole-Licofelone on *SAP* Gene Expression Levels

**FIGURE 5:**
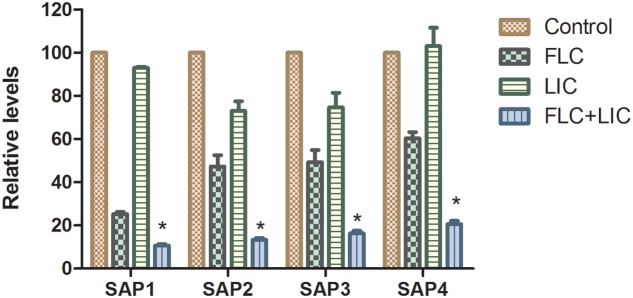
Relative gene expression levels of *SAP1*, *SAP2*, *SAP3*, and *SAP4* in resistant *C. albicans* (CA10). Cells were treated with fluconazole (1 μg/mL), licofelone (16 μg/mL) and their combination. Total RNA was extracted and reverse transcribed to cDNA for further real-time quantitative PCR to detect gene expression levels. ^∗^*P* < 0.05 compared to the control, fluconazole alone and licofelone alone group. The experiment was repeated on 3 independent occasions (*n* = 3). Values represent the means standard deviations from three replicates.

To investigate the effects of licofelone and fluconazole on resistant *C. albicans* (CA10) secreted aspartyl proteinase activities, the expression of 4 *SAP* family genes was analyzed by RT-PCR. The results showed that both licofelone combined with fluconazole group and fluconazole alone group can down-regulate the expression levels of *SAP1*, *SAP2*, *SAP3*, and *SAP4* compared to the control group (**Figure 5**). The level of decreased expression in the combination group was more remarkable than the fluconazole alone group (*P* < 0.05). The expression level of *SAP1* in the fluconazole alone group was significantly decreased compared to the control group (*P* < 0.05). While the expression of *SAP1* to *SAP4* in licofelone alone group shows no obvious difference compared to the control group.

### Effect of Fluconazole-Licofelone on *RAS1*, *CYR1*, *TPK2, EFG1*, *BCR1*, *ALS1*, *ALS3* and *HWP1* Gene Expression Levels

**FIGURE 6:**
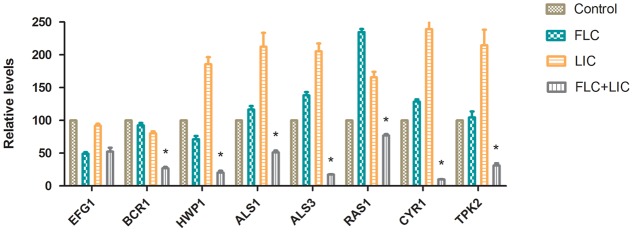
Relative gene expression levels of *RAS1*, *CYR1*, *TPK2*, *EFG1*, *BCR1*, *HWP1, ALS1* and *ALS3* in resistant *C. albicans* (CA10). Cells were treated with fluconazole (1 μg/mL), licofelone (16 μg/mL) and their combination. Total RNA was extracted and reverse transcribed to cDNA for further real-time quantitative PCR to detect gene expression levels. ^∗^*P* < 0.05 compared to the control, fluconazole alone and licofelone alone group. The experiment was repeated on 3 independent occasions (*n* = 3). Values represent the means standard deviations from three replicates.

To further investigate the effects of licofelone and fluconazole on resistant *C. albicans* (CA10) biofilm development, the expression levels of 8 biofilm-related genes were analyzed by RT-PCR. The results showed that the combination of licofelone with fluconazole group down-regulated the *RAS1*, *CYR1*, *TPK2*, *BCR1*, *HWP1*, *ALS1* and *ALS3* expression in comparison with control group (**Figure 6**). The combination group significantly decreased the expression level of *RAS1* (3-fold), *CYR1* (10-fold), *TPK2* (3-fold), *BCR1* (4-fold), *HWP1* (3-fold), *ALS1* (2-fold) and *ALS3* (8-fold) compared to the fluconazole group (*P* < 0.05). While the expression of *RAS1*, *CYR1*, *TPK2*, *ALS1*, *ALS3*, and *HWP1* expression on licofelone alone were higher than fluconazole alone group. However, the down-regulation of *EFG1* expression has no difference between the drug combination group and the fluconazole group (*P* > 0.05). In addition, both groups decreased *EFG1* expression relative to the growth control group.

### Effect of Fluconazole-Licofelone on *C. albicans* Morphogenesis

**FIGURE 7:**
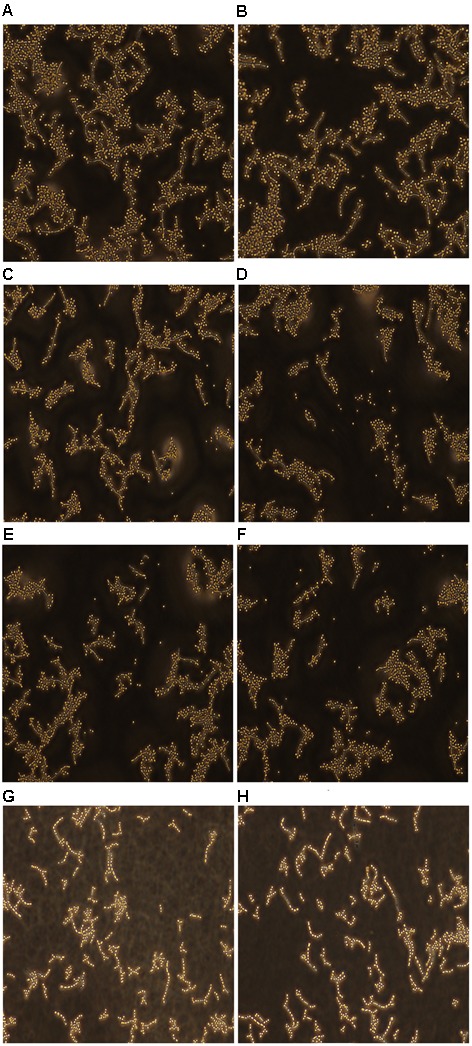
Fluorescence microscope examined the yeast-filament transition of resistant *C. albicans* (CA10). After 24 h of incubation, *C. albicans* treated with different drugs as described. **(A,B)** Untreated controls group; **(C,D)** fluconazole treated (1 μg/mL) group; **(E,F)** licofelone treated (16 μg/mL) group; **(G,H)** the combination of fluconazole (1 μg/mL) and licofelone (16 μg/mL) treated group. The experiment was repeated on 3 independent occasions.

The hyphal formation was observed by fluorescence microscope. The images of resistant *C. albicans* (CA10) showed that the filamentation was reduced with the presence of licofelone combined with fluconazole (**Figures 7G,H**) compared to the fluconazole treated group (**Figures 7C,D**) and control group (**Figures 7A,B**). There were entirely yeast cells and no filaments in the combination group. In the control group, there were a large area of hypha gather together, while in the fluconazole or licofelone alone group, there were still some hypha gathered, but it was much lesser than the control group. This may be caused that the two-drug combination can decrease the filament formation.

### Effect of Licofelone on Drug Efflux Pumps Activity of Resistant *C. albicans*

**FIGURE 8:**
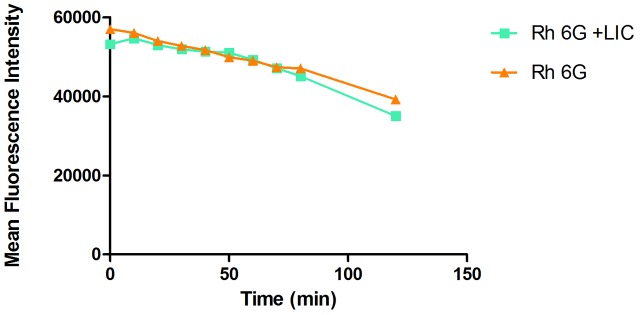
The effect of licofelone on the efflux of Rh6G in resistant *C. albicans*. The efflux of Rh6G in the absence and presence of licofelone (16 μg/mL) were determined by a flow cytometer. MFIs represent the intracellular Rh6G in *C. albicans*. ^∗^*P* > 0.05 compared to the Rh6G alone group. The experiment was repeated on 3 independent occasions.

The rhodamine 6G assay results showed that the licofelone group and the growth control group have the same decline tendency (**Figure 8**). No significant differences between groups were observed (*P* > 0.05).

## Discussion

Therapies such as the use of novel compounds combined with the azoles could be an effective solution for candida infections, as it can expand antibiotic spectrum, improve antifungal efficacy, and reduce side effects (Shrestha et al., 2015; Gu et al., 2016). Several *in vitro* and *in vivo* studies demonstrated that COX inhibitors, for instance, ibuprofen, aspirin, and indomethacin have certain antifungal activities by suppressing *C. albicans* PGE_2_ production and biofilm formation (Alem and Douglas, 2005; Bink et al., 2012; Liu et al., 2014). The combination of ibuprofen and fluconazole can enhance fluconazole susceptibly to *C. albicans* by decreasing the MIC of fluconazole (Costa-de-Oliveira et al., 2015). mPGES-1, as the terminal enzyme downstream of COX-2 can catalyze the PGE_2_ biosynthesis with fewer side effects, ideally not affecting the formation of other housekeeping PGs (Vidal et al., 2007; Koeberle et al., 2010). Various pharmacodynamic studies have confirmed that a novel dual mPGES-1/LOX inhibitor, licofelone was effective in many types of diseases and has anti-asthmatic and anticonvulsant effects (Rotondo et al., 2002; Kulkarni and Singh, 2007). The simultaneous inhibitions of both enzymes enable licofelone to have a superior anti-inflammatory effect and obviate the gastrointestinal side-effects compared to the NSAIDS. This better safety profile has been authenticated in healthy individuals (Charlier and Michaux, 2003; Bias et al., 2004). However, weather licofelone has the similar antifungal activity as COX inhibitor has not been elucidated. In this study, we first evaluated the effect of fluconazole combined with licofelone against resistant *C. albicans in vitro*, licofelone alone has a moderately great effect on resistant *C. albicans*: the MIC of licofelone alone against resistant *C. albicans* was 128 μg/mL, and it can synergistically work with fluconazole: the MIC_80_ of fluconazole was decreased from 512 to 1 μg/mL when combined with licofelone (16 μg/mL), while the combination has little effect on sensitive *C. albicans* and non-albicans strains (**Table 1**). Secondly, the combined antifungal effect of fluconazole-licofelone against *C. albicans* biofilm cells was observed in different time point. The two-drug combination significantly reduced *C. albicans* biofilm formation over 12 h, but after 24 h, there was almost no anti-biofilm effect observed (**Table 2**). The *C. albicans* biofilm formation has three phases: adherence stage (0–11 h) in which the fungal pathogens adhere to the surface, development stage (12–30 h) in which the carbohydrate-rich extracellular matrix (ECM) are produced, and maturation stage (31–72 h) in which the biofilm structure is held together by an ECM. The resistance of biofilms to antimicrobial agents is often attributed to its failure to pass the biofilm matrix (Al-Fattani and Douglas, 2006). This may be the reason why the antifungal effect of the combination can only inhibit biofilm over the process of formation, but has no inhibiting effect on the mature biofilm. Further, for the future experiments, we will focus on the effect and antifungal mechanisms of resistant *C. albicans*.

In addition, *G. mellonella* model recently has been used to study fungal virulence and antifungal drug activity, since it has a similar immune response to mammals (Vilcinskas, 2011; Favre-Godal et al., 2014), and the correlation between virulence of *C. albicans* mutants in mice and *G. mellonella* model has been confirmed (Brennan et al., 2002). It is worth mentioning that, with these advantages and the low cost, lack of ethical concerns and easy manipulation, *G. mellonella* model has gained wide acknowledgment. As documented in our *in vivo* study, the combination of fluconazole with licofelone in *G. mellonella* infected model significantly improved the survival rate of larvae compared to the fluconazole treated group (*P* < 0.05). The death rate of the larvae is a dose dependent (**Figure 2**), as previously described (Scorzoni et al., 2013). To complement the studies *in vivo*, we further performed the fungal burden determination (**Figure 3**) and microscopic observation (**Figure 4**) in larvae after infection. Our results confirmed the correlation of the virulence with the degree of damage on the histological tissue of *G. mellonella* with fewer CFUs was observed in the combined group.

*Candida albicans* biofilm is a complex three-dimensional architecture with an abundant extracellular matrix. It is known to enhance the fungal resistance to most commonly used antifungal agent and the ability of cells to adhere is indispensable for all stages of *C. albicans* biofilm formation (Chandra et al., 2001; Nobile and Johnson, 2015). Earlier evidence only indicated that the effectiveness of COX inhibitors against *C. albicans* is based on their activity of inhibiting biofilm formation by decreasing PGE_2_ production. Here we investigated the further antifungal mechanisms of licofelone combined with fluconazole. RAS/cAMP/PKA pathway is important for *C. albicans* virulence by regulating hyphal growth (Rocha et al., 2001). In this pathway, the activation of Ras1 can lead to the generation of a cAMP signal that promotes PKA-mediated activation of the transcriptional regulator proteins *EFG1* and *BCR1*, which that play an essential role in regulating the morphogenesis and expression level of cell wall-associated adhesion gene such as *ALS1*, *ALS3*, and *HWP1* that are associated with morphogenetic response (Nobile and Mitchell, 2005). Our *in vitro* result showed that licofelone combined with fluconazole has great effects on inhibiting biofilm formation in different stages. Regarding how the drug combination influences the biofilm formation, the results showed that the combination has no influence on *EFG1* expression level, but dramatically down-regulated the expression of *BCR1* (**Figure 6**), suggesting that the possibility of drug combination against resistant *C. albicans* biofilm is through the regulation of the central regulator *BCR1*, not by *EFG1*. We further examined the adhesion-related genes *HWP1*, *ALS1* and *ALS3* expression levels in the presence of the combination. We found that the expression level of those genes was decreased 3-fold, 2-fold, and 8-fold, respectively, compared to the fluconazole group (*P* < 0.05) (**Figure 6**). Mutational analysis indicated that strains lacking all functional *HWP1*, *ALS1* and *ALS3* alleles (*hwp1*Δ*/hwp1*Δ *als1*Δ*/als1*Δ *als3*Δ*/als3*Δ) were not able to generate adherent cells in biofilm models (Nobile et al., 2008). Thus, our results demonstrated that the fluconazole combined with licofelone may reduce the biofilm formation of *C. albicans* by inhibiting adhesion, and this inhibition is partly due to the down-regulation of biofilm cell wall adhesion-related genes *ALS1*, *ALS3* and *HWP1*. These results were further confirmed by florescence microscope analysis. The two-drug combination-treated strains produced a biofilm with only yeast cells and little filament, but the filamentation was more abundant with the presence of fluconazole group and the growth control group (**Figure 7**). Moreover, licofelone combined with fluconazole decreased the expression level of *RAS1*, *CYR1* and *TPK2*, indicating that the inhibition of biofilm-specific related genes is probably through RAS/cAMP/PKA pathway (**Figure 6**). Previous studies have also shown that secreted aspartyl proteinase and phospholipases were believed to be important factors involved in the pathogenicity and virulence of *C. albicans* (Ghannoum, 2000; Feng et al., 2016). These hydrolytic enzymes were secreted by the fungus that lead to the colonization and infections (Nailis et al., 2010). Secreted aspartyl proteinases are related to the adhesion and hydrolytic activity in mucosal tissue (Ramage et al., 2012). The study by Mendes et al. has indicated that biofilms of *C. albicans* secreted more Saps than planktonic cells (Mendes et al., 2007), and Saps are known to be intrinsically associated with the hyphal phase of *C. albicans* (Chen et al., 2002; Felk et al., 2002). We also examined the SAP gene expression by RT-PCR. The combination of fluconazole and licofelone significantly down-regulated the *SAP1*-*SAP4* expression levels compared to fluconazole group (*P* < 0.05) (**Figure 5**). In terms of extracellular phospholipase, the egg yolk agar plate assay showed that licofelone combined with fluconazole decreased the phospholipase activity at low concentration compared to the fluconazole group, and the inhibition effect was positively correlated to the drug concentration (**Table 3**). Furthermore, licofelone combined with fluconazole has no effect on the drug efflux pumps compared to the fluconazole group (*P* > 0.05) (**Figure 8**), thus the antifungal activity of the combination was irrelevant to reverse the mechanism of drug efflux. To our knowledge, the present study provides an substantial advance over recent studies of ours and in the field by first finding that the synergistic effect of licofelone combined with fluconazole against resistant *C. albicans* in both *in vitro* and *in vivo*. The underlying mechanisms are mainly explained by attenuation of virulence factor such as secreted aspartyl proteinase, reduction of extracellular phospholipase activity, and inhibition of the transition between yeast and hyphal growth forms, but not to affect the drug efflux pumps activity. These results indicate that licofelone could be a favorable antifungal agent and a promising synergist with fluconazole against resistant *C. albicans*, and more in-depth mechanisms needed to be elucidated.

## Author Contributions

XL, TL, DW, WS, YY, JL, and SS performed the experiments. XL and SS designed the research. XL analyzed the data and wrote the paper. All authors approved the manuscript for publication.

## Conflict of Interest Statement

The authors declare that the research was conducted in the absence of any commercial or financial relationships that could be construed as a potential conflict of interest.
