# Supplementary material for: Synergistic Antifungal Effect of Fluconazole Combined with Licofelone against Resistant Candida albicans
Source: Front Microbiol. 2017 Nov 7;8:2101. doi: 10.3389/fmicb.2017.02101 (PMC5681995; doi:10.3389/fmicb.2017.02101)
Supplement: Supplementary file 1 [file Table_1.pdf]

Gene	Sequence (5' - 3')	Reference
ACT1-f	AAGAATTGATTTGGCTGGTAGAGA	This work
ACT1-r	TGGCAGAAGATTGAGAAGAAGTTT	This work
SAP1-f	AAAGACAAGCCCTCCCAGTT	This work
SAP1-r	GCATCAGGAACCCATAAATCA	This work
SAP2-f	TCCAAGTGGTTCATCAGCTTC	This work
SAP2-r	ATCGAAACACCACCAAATCC	This work
SAP3-f	TTCTCCAGGGTTTGTTGCTT	This work
SAP3-r	TCTCTTGACGTTGACGTTGG	This work
SAP4-f	ACCGTTGGTATTGGTGGTGT	This work
SAP4-r	GAGTCCTGGTGGCTTCGTT	This work
HWP1-f	GCTCAACTTATTGCTATCGCTTATTACA	(1)
HWP1-r	GACCGTCTACCTGTGGGACAGT	(1)
ALS1-f	GACTAGTGAACCAACAAATACCAG	(2)
ALS1-r	ACCAGAAGAAACAGCAGGTG	(2)
ALS3-f	CCAAGTGTTCCAACAACTGAA	(2)
ALS3-r	GAACCGGTTGTTGCTATGGT	(2)
BCR1-r	GAGCACGCATCTATGGCTTA	(3)
BCR1-f	GGCTGTCCATGTTGTTGTTG	(3)
RAS1-r	GTCTTTCCATTTCTAAATCAC	(4)
RAS1-f	GGCCATGAGAGAACAATATA	(4)
CYR1-r	TCTTGAACTGCCAGACGATG	(5)
CYR1-f	CCAACAAACGACCAAAAGGT	(5)
TPK2-r	CCAACAAACGACCAAAAGGT	(4)
TPK2-f	AGAAACTTCACATCACCAAG	(4)
EFG1-r	GGGTGAAGGGTGAACTGAAC	This work
EFG1-f	CCAACAGCAACAACAAAAGC	This work

Table 1 :Real time PCR primers used in this study

1. **Rajendran R, Sherry L, Lappin DF, Nile CJ, Smith K, Williams C, Munro CA, Ramage G.** 2014. Extracellular DNA release confers heterogeneity in Candida albicans biofilm formation. BMC Microbiol **14:**303.
2. **Roudbarmohammadi S, Roudbary M, Bakhshi B, Katiraee F, Mohammadi R, Falahati M.** 2016. ALS1 and ALS3 gene expression and biofilm formation in Candida albicans isolated from vulvovaginal candidiasis. Adv Biomed Res **5:**105.
3. **Alves CT, Silva S, Pereira L, Williams DW, Azeredo J, Henriques M.** 2014. Effect of progesterone on Candida albicans vaginal pathogenicity. Int J Med Microbiol **304:**1011-1017.
4. **Li Y, Chang W, Zhang M, Ying Z, Lou H.** 2015. Natural product solasodine-3-O-beta-D-glucopyranoside inhibits the virulence factors of Candida albicans. FEMS Yeast Res **15**.
5. **Hsu CC, Lai WL, Chuang KC, Lee MH, Tsai YC.** 2013. The inhibitory activity of linalool against the filamentous growth and biofilm formation in Candida albicans. Med Mycol **51:**473-482.
